# Specificity of brassinosteroid perception and its integration at signaling crossroads in vascular development

**DOI:** 10.1111/tpj.70570

**Published:** 2025-11-14

**Authors:** Noel Blanco‐Touriñán, Christian S. Hardtke

**Affiliations:** ^1^ Department of Plant Molecular Biology University of Lausanne Lausanne Switzerland; ^2^ Instituto de Biología Molecular y Celular de Plantas, Consejo Superior de Investigaciones Científicas—Universitat Politècnica de València 46022 Valencia Spain

**Keywords:** brassinosteroid, receptor kinases, plant growth, vascular development, CLE peptides

## Abstract

Brassinosteroids (BRs) are key regulators of plant growth and development. The BR receptor BRASSINOSTEROID‐INSENSITIVE 1 (BRI1) is broadly expressed across root tissues, yet classical genetic studies suggested that BR signaling can also act non‐cell‐autonomously. Nevertheless, recent advances—based on tissue‐specific CRISPR, state‐of‐the‐art imaging and single‐cell transcriptomics—reveal that BR perception operates largely cell‐autonomous and strongly context‐dependent. Here, we also review emerging evidence demonstrating that BR signaling is spatially confined and mechanistically integrated with other signaling pathways to regulate tissue‐specific developmental outcomes. Rather than acting as a uniform growth trigger, BR functions as a modular, locally tuned regulator coordinating plant development via both canonical and non‐canonical pathways.

## Significance Statement

Recent advances demonstrate that brassinosteroid perception occurs largely cell‐autonomously and is tightly shaped by tissue context. This refined view highlights brassinosteroid signaling as a locally tuned, modular system integrated with other pathways to direct specific developmental outcomes.

## INTRODUCTION

Brassinosteroids (BRs) are essential plant steroid hormones that regulate plant growth, developmental plasticity, and stress responses. BR biosynthesis takes place, at least in part, in the endoplasmic reticulum, as BR biosynthesis enzymes in the model plant *Arabidopsis thaliana* (Arabidopsis) have been found to localize in this organelle (Nolan et al., 2020). Once synthesized, BRs are transported from the cytosol to the apoplast by the ATP‐binding cassette transporters ABCB1 and ABCB19 (Wei et al., 2025; Ying et al., 2024). BR perception initiates at the cell surface through a well‐studied signaling pathway. BRs bind to the extracellular domains of the leucine‐rich repeat (LRR) receptor kinase BRASSINOSTEROID‐INSENSITIVE 1 (BRI1) and its redundant homologs BRI1‐LIKE 1 (BRL1) and BRL3 (Caño‐Delgado et al., 2004; Hothorn et al., 2011; Kinoshita et al., 2005; She et al., 2011; Wang et al., 2001; Zhou et al., 2004). This binding triggers the association of BRI1 with its co‐receptor BRI1 ASSOCIATED KINASE 1 (BAK1; also known as SOMATIC EMBRYOGENESIS RECEPTOR KINASE 3, SERK3) (Li et al., 2002; Nam & Li, 2002; Wang et al., 2008). The formation of this active BRI1‐BAK1 complex displaces inhibitory proteins—BRI1 KINASE INHIBITOR 1 (BKI1) from BRI1 and BAK1‐INTERACTING RECEPTOR‐LIKE KINASE 3 (BIR3) from BAK1—and enables trans‐phosphorylation of their cytoplasmic kinase domains, activating BRI1 kinase activity (Imkampe et al., 2017; Jaillais et al., 2011; Wang & Chory, 2006). Activated BRI1 then phosphorylates BR‐SIGNALING KINASE (BSK) and CONSTITUTIVE DIFFERENTIAL GROWTH (CDG) kinases, which in turn activate BRI1 SUPPRESSOR (BSU) phosphatases (Kim et al., 2011; Mora‐García et al., 2004; Muto et al., 2004; Tang et al., 2008). BSUs promote BR signal transduction by dephosphorylating and inactivating the key negative BR regulator BRASSINOSTEROID‐INSENSITIVE 2 (BIN2), a member of the GLYCOGEN SYNTHASE KINASES 3 (GSK3) family (Choe et al., 2002; He et al., 2002; Kim et al., 2011; Li et al., 2001; Li & Nam, 2002). In the absence of BRs, BIN2 phosphorylates the transcription factors BRASSINOSTEROID‐INSENSITIVE EMS SUPPRESSOR 1 (BES1) and BRASSINAZOLE RESISTANT 1 (BZR1), and their homologs (BEH1‐4), thereby reducing their activity by promoting their degradation, inhibiting their DNA binding and facilitating their nuclear exclusion (Gampala et al., 2007; He et al., 2002, 2005; Kim et al., 2024; Nolan et al., 2020; Ryu et al., 2007; Wang et al., 2021; Yin et al., 2002). Upon BR perception, protein phosphatase 2A (PP2A) dephosphorylates BES1/BZR1, allowing their accumulation in the nucleus, where they regulate BR‐responsive gene expression (Tang et al., 2011). The numerous genes controlled by these transcription factors include BR biosynthesis pathway genes, establishing feedback regulation and hormone homeostasis (Sun et al., 2010; Tanaka et al., 2005; Yu et al., 2011) (see Nolan et al. [2020] for a comprehensive review of BR signaling).

In addition to the canonical pathway, evidence suggests the existence of non‐canonical BR signaling pathways (Fàbregas et al., 2018; Tanaka et al., 2009; Teng et al., 2023). For example, in rice, the heterotrimeric G‐protein alpha subunit RGA1 (encoded by the *D1* gene) functions independently of OsBRI1, promoting certain BR responses through a not yet fully understood mechanism (Wang et al., 2006). Similarly, in Arabidopsis, non‐canonical BR signaling mechanisms have been proposed in vascular development (see elaboration below on the mechanism involved) as well as under non‐physiological conditions. For instance, under drought conditions, *bes1* ^*D*^ gain‐of‐function mutants exhibit drought hypersensitivity (Ye et al., 2017), whereas overexpression of the vascular BRL3 receptor promotes drought resistance (Fàbregas et al., 2018). This suggests that BRL3 may function independently of the canonical growth‐promoting BRI1 pathway in such contexts (Fàbregas et al., 2018). Moreover, increasing evidence indicates that distinct BZR1 homologs can have divergent functions within the same biological process (Ammitsøe et al., 2025), highlighting the importance of investigating the roles of the different components of BR signaling in a context‐dependent manner.

Extending this context dependency to the cellular scale, the question arises as to how BR signaling operates within specific tissues and cell types? BR receptors show distinct expression patterns: unlike BRI1, which is widely expressed throughout the root tissues, BRL1 and BRL3 are predominantly enriched in the vasculature (Caño‐Delgado et al., 2004; Friedrichsen et al., 2000; Zhou et al., 2004). Meanwhile, the master regulators BZR1 and BES1 exhibit uniform expression in the root tip, suggesting that BR signaling may function across multiple tissues (Chaiwanon & Wang, 2015). Paradoxically, BR signaling may act non‐cell‐autonomously however (Fridman et al., 2014; Graeff et al., 2020; Hacham et al., 2011; Hategan et al., 2014; Kang et al., 2017; Savaldi‐Goldstein et al., 2007; Vragović et al., 2015), although it can also function cell‐autonomously in certain contexts (Kang et al., 2017; Lozano‐Elena et al., 2018). In cell‐autonomous signaling, the same cell that perceives BR also initiates the downstream response. In contrast, non‐cell‐autonomous signaling occurs when BR perception in one cell triggers responses in neighboring (or even distant) cells. The most extensively studied example of BR signaling is the non‐cell‐autonomous regulation of shoot and root growth (Graeff et al., 2020; Hacham et al., 2011; Savaldi‐Goldstein et al., 2007) (see below). Intriguing examples of spatially restricted BR signaling within individual organs have also emerged. For instance, in leaves, BRs act in the epidermis to promote air space morphogenesis non‐cell‐autonomously in the mesophyll cells (Fitzsimons et al., 2025). In some other developmental contexts, however, spatial coordination does not involve a signal derived from BR signaling *per se*. During endosperm development, for example, BR perception in the maternal seed coat regulates cell‐wall‐related processes that determine the mechanical properties of the seed coat (Lima et al., 2024). These physical constraints imposed by the seed coat influence endosperm proliferation (Lima et al., 2024). As mentioned, BR signaling also functions cell‐autonomously in certain contexts. This is exemplified by their role in regulating protophloem differentiation (Kang et al., 2017) (see below) and maintaining the stem cell niche (Lozano‐Elena et al., 2018). In the latter case, BR perception by BRI1 activates the canonical signaling pathway involving BES1, which cell‐autonomously promotes quiescent center divisions (Lozano‐Elena et al., 2018). This mechanism appears to be essential for stem cell replenishment following tissue damage.

In the first part of this review, we will revisit the concept of non‐cell‐autonomous BR signaling in roots and shoots. In the second part, we will focus on the molecular mechanisms of cell‐autonomous BR signaling, using vascular development as a model to illustrate pathway specificity and its interactions with other signaling networks.

## LESSONS ON THE SPECIFICITY OF BR SIGNALING FROM GENETIC STUDIES

**Figure 1:**
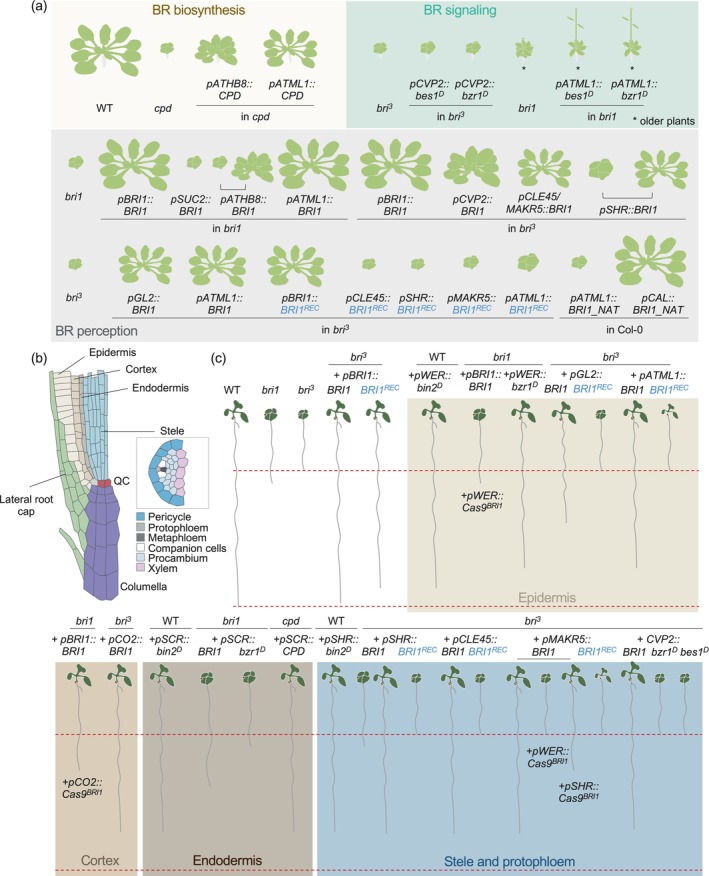
Summary of findings supporting non‐cell autonomous and cell‐autonomous brassinosteroid (BR) signaling. (a) Representation of shoot phenotypes of plants expressing BR biosynthetic genes, signaling effectors or receptors under different tissue‐specific promoters in different genetic backgrounds. *bri* ^*3*^ = *bri1 brl1 brl3* triple mutant. * indicates older plants relative to the others shown. Phenotypes are approximate, as not all plants or mutant backgrounds were analyzed in parallel. When multiple conclusions were reported (e.g., BRI1 expressed under *ATHB8* and *SHR* promoters in *bri1* or *bri* ^*3*^, respectively), both are depicted. (b) Longitudinal view of an Arabidopsis root meristem. For the stele, comprising the pericycle and vasculature, a cross‐section is shown. QC, quiescent center. (c) Schematic of root phenotypes in plants with tissue‐specific CRISPR/Cas9‐mediated gene knockouts or expression of BR signaling effectors or receptors in different backgrounds. Phenotypes are approximate, as not all genotypes were tested in parallel. Shoot phenotypes here may not reflect reality, as they are not always described in the literature.

A long‐standing mystery in the field has been the nature and underlying mechanisms of the proposed non‐cell‐autonomous effects of BR perception. This idea originated from complementation studies of the Arabidopsis *bri1* mutant, where expression of BRI1 driven by the epidermis‐specific promoter *ARABIDOPSIS THALIANA MERISTEM LAYER1* (*ATML1*) was sufficient to rescue the shoot dwarf phenotype (Savaldi‐Goldstein et al., 2007) (see phenotypes in Figure 1a). It was later shown that this complementation also occurs in the loss‐of‐function mutant lacking all three BR receptors (*bri1 brl1 brl3*) and when using other epidermal‐specific promoters such as *GLABRA2* (*GL2*), suggesting that BR signaling could act beyond the cells where it is perceived (Kang et al., 2017; Vragović et al., 2015). This idea is supported by complementation studies in which expression of the BR biosynthetic gene *CONSTITUTIVE PHOTOMOPHOGENESIS AND DWARF* (*CPD*) under an epidermis‐specific promoter was sufficient to rescue the dwarf phenotype of *cpd* mutants (Savaldi‐Goldstein et al., 2007). In contrast, expression of BRI1 under the inner tissue‐specific promoters *ARABIDOPSIS THALIANA HOMEOBOX 8* (*ATHB8*; provascular) or *SUCROSE‐PROTON SYMPORTER 2* (*SUC2*; phloem companion cells) very weakly complemented the *bri1* mutant defects (Hategan et al., 2014; Savaldi‐Goldstein et al., 2007). In line with this, recent findings indicate that overexpression of a natural antisense transcript (NAT) for *BRI1* under an epidermal promoter induces *bri1*‐like phenotypes with reduced *BRI1* expression, whereas NAT expression in sub‐epidermal tissues (with the *PHOSPHOENOL PYRUVATE–CARBOXYLASE* [*pCAL*] promoter) produces wild‐type‐like plants (Bhasin et al., 2024) (Figure 1a). Together, these data support a model in which, in the shoot, a non‐cell autonomous signal originating from the epidermis triggers growth in the underlying ground tissues.

Recently, this model has been challenged by observations showing that expression of BRI1 or its homologs under phloem‐ (e.g., *COTYLEDON VASCULAR PATTERN 2* [*CVP2*], *CLAVATA 3/EMBRYO SURROUNDING REGION‐RELATED 45* [*CLE45*] or *MEMBRANE‐ASSOCIATED KINASE REGULATOR 5* [MAKR5]) or stele‐specific promoters (e.g., *SHORT ROOT* [*SHR*]) can also complement the dwarf defects in the *bri1 brl1 brl3* triple mutant (Blanco‐Touriñán et al., 2024; Graeff et al., 2020; Kang et al., 2017) (see Figure 1a–c). Moreover, epidermis‐specific expression of dominant versions of BES1 (bes1^D^) or BZR1 (bzr1^D^) at best partially rescues the shoot and root growth defects of the *bri1* single mutant (Figure 1a,c), whereas the *bzr1* ^*D*^ or *bes1* ^*D*^ gain‐of‐function mutants almost fully rescue *bri1* defects (Chaiwanon & Wang, 2015). Complementary evidence comes from studies on BR biosynthesis: expression of CPD under the provascular *ATHB8* promoter partially rescues the *cpd* mutant phenotype (Savaldi‐Goldstein et al., 2007) (Figure 1a). These findings suggest that BR biosynthesis and perception within inner tissues also contribute to systemic growth regulation. Remarkably, this regulation is not limited to normal physiological conditions but also occurs under stress, such as elevated temperatures. Under such conditions, BRL3‐dependent signaling in phloem companion cells promotes growth by activating BES1 and regulating hormone homeostasis and central carbon metabolism (Gupta et al., 2023). This suggests that phloem companion cells also generate systemic signals that relay to the epidermis to coordinate plant growth under stress. In summary, all these observations indicate that BR perception can act non‐cell‐autonomously in both directions—from outer to inner tissues and vice versa—highlighting the complexity and spatial integration of BR signaling in coordinating plant growth (see also Aardening et al. [2025] for a comprehensive review of cell type‐specific perturbations of BR signaling and biosynthesis components).

Adding another layer of complexity, translatome analyses revealed context‐specific effects of BR signaling on gene expression and a more intricate form of inter‐cell layer communication. Specifically, epidermal BR perception activates auxin‐related genes to promote cell division in the meristem, while BR perception in the stele indirectly restrains this effect by controlling BR biosynthesis, suggesting a coordinated interplay between tissues that influence each other (Vragović et al., 2015). But what exactly is the mobile signal that enables BR signaling to act beyond the cells where it is perceived and thus mediates coordination between tissues? Because BRs are hydroxylated steroid hormones thought to be membrane‐impermeable, it seems unlikely that these hormones themselves move between cells (Symons & Reid, 2004). Recent work however has shown that plasmodesmata mediate the passage of BRs between neighboring cells (Wang et al., 2023). Yet, this mechanism cannot account for movement between distant tissues, as would be expected for non‐cell‐autonomous signaling. For epidermis‐derived regulation affecting other tissues in roots, one model proposes that BR‐induced auxin may act as a mobile, non‐cell‐autonomous signal promoting stem cell division and delaying differentiation in the meristem (Vragović et al., 2015). Mechanical cues that emerge from the interconnected walls of the cells could also contribute: for instance, BR‐dependent, cell type‐specific regulation of the cell wall may modulate radial growth of the root meristem (Fridman et al., 2021). In line with this, in stems, the non‐cell‐autonomous regulation relies on mechanical changes, with epidermal BR signaling locally reducing epidermal mechanical constraints and enabling the internal tissues to expand (Kelly‐Bellow et al., 2023). Recent advances in single‐cell mRNA sequencing, high‐resolution imaging and tissue‐specific genome editing suggest alternative (and complementary) mechanisms.

## INSIGHTS FROM SINGLE‐CELL mRNA SEQUENCING AND TISSUE‐SPECIFIC CRISPR

Emerging technologies have provided new insights into the spatial complexity of BR signaling. Single‐cell mRNA sequencing of Arabidopsis roots has shown that BRs regulate distinct gene sets in different spatiotemporal contexts (Nolan et al., 2023). Both the epidermis and the elongating cortical cells are highly responsive to BR, highlighting that tissue‐specific BR signaling contributes unequally to overall organ growth. This aligns with the observations from tissue‐specific CRISPR knockouts in complemented *bri1* single mutants expressing BRI1‐GFP under its native promoter. Epidermis‐specific knockout of the *BRI1* transgene using the *WEREWOLF* (*WER*) promoter reverses the rescue phenotype (see root phenotypes in Figure 1c), supporting the model that the epidermis non‐cell‐autonomously regulates root growth (Nolan et al., 2023). However, cortex‐specific knockout of *BRI1* in the same line also reduces root growth, despite normal levels of epidermal BRI1 signal (Nolan et al., 2023). In this tissue, BR signaling triggers the expression of cell wall‐related genes during cell transition from proliferation to elongation (Nolan et al., 2023). More intriguingly, in a complemented *bri1 brl1 brl3* triple mutant line expressing BRI1‐CITRINE under a phloem‐specific promoter, epidermis‐specific knockout via the *WER* promoter phenocopies *bri1 brl1 brl3* roots (Blanco‐Touriñán et al., 2024). Conversely, knockout of the protophloem expression in this line using the *SHR* promoter reduces root growth but to an intermediate extent, with roots longer than those of the triple mutant (Blanco‐Touriñán et al., 2024). These observations suggest that BR perception in multiple tissues contributes to (root) organ growth, but they also raise a compelling question: how can BRI1 expression driven by a protophloem‐specific promoter rescue root growth in the triple mutant, yet, this rescue is reversed by knocking out *BRI1* in the epidermis?

The answer, at least in part, is likely that confocal microscopy consistently reveals a weak epidermal BRI1 signal regardless of the promoter driving expression (Blanco‐Touriñán et al., 2024). Single‐cell mRNA sequencing confirms that the BRI1‐CITRINE fusion protein driven by either protophloem‐ or epidermis‐specific promoters is detected in multiple cell types (Blanco‐Touriñán et al., 2024). Moreover, the loss of BR signaling in the tissue targeted by the promoter did not prevent BRI1 accumulation in other cell types (Blanco‐Touriñán et al., 2024). This suggests that the broad expression pattern is not due to the movement of *BRI1* mRNA or BRI1 protein but rather reflects promoter activity or features of the gene body influencing expression. Notably, promoter activity itself does not change in response to BRI1‐CITRINE expression (Blanco‐Touriñán et al., 2024). An elegant experiment further clarifies this point: when a recoded version of the *BRI1* gene—engineered with synonymous codon changes but encoding the identical protein—was expressed under tissue‐specific promoters (e.g., epidermis and protophloem), its expression was strictly confined to the targeted tissue, and it failed to rescue the root growth defects of the *bri1 brl1 brl3* triple mutant (Blanco‐Touriñán et al., 2024) (Figure 1c). These findings lead to two important conclusions: (1) BR signaling is required in multiple tissues to fully restore normal root growth in *bri1 brl1 brl3* mutants, and (2) non‐cell‐autonomous BR signaling between tissues is unlikely to be the primary mechanism regulating root and shoot growth. The idea of cell‐autonomous BR action in root growth aligns with previous observations showing that perturbing BR signaling in specific tissues can impact overall primary root growth (Ackerman‐Lavert et al., 2021). For example, targeting inhibition of BR signaling in wild‐type plants through ectopic expression of a dominant version of BIN2 (*bin2‐1*) under tissue‐specific promoters—such as *WER* (epidermis), *SCARECROW* (*SCR*; endodermis and quiescent center) and *SHR* (stele)—consistently led to reduced primary root growth (Ackerman‐Lavert et al., 2021) (Figure 1c). Despite this shared outcome at the organ level, the underlying cellular phenotypes differ between tissues. Expression of *bin2‐1* in the outer layers produced a *bri1*‐like meristem, characterized by wide meristems with short cells, indicative of low BR signaling (Ackerman‐Lavert et al., 2021). In contrast, *SHR:bin2‐1* roots displayed the opposite phenotype: a narrower meristem with elongated meristematic cells, resembling a high‐BR response (Ackerman‐Lavert et al., 2021). It is important to consider however that BIN2 functions beyond BR signaling, acting as a central hub in multiple signaling pathways (Kim et al., 2023). Therefore, potential indirect effects cannot be excluded in these experiments. To more specifically dissect BR‐dependent responses, combining tissue‐specific CRISPR with recoded BRI1 or dominant‐active forms of BES1 and BZR1 in BR‐blind plants offers a cleaner strategy to investigate the tissue‐specific contributions and cell‐autonomous aspects of BR signaling.

The spatial complexity of BR responses extends beyond receptor localization to include tightly regulated biosynthesis and hormone distribution across root tissues. BRs are synthesized through a multi‐step pathway involving enzymes that are not uniformly expressed throughout the root tissues (Vukašinović et al., 2021). Thus, not all cells in the Arabidopsis root possess the complete biosynthetic machinery, and the completion of BR biosynthesis depends on the cell‐to‐cell movement of hormone precursors (Vukašinović et al., 2021). This clearly suggests that the *BRI1* gene safeguards the ability of individual cells to respond to differential BR cues (Blanco‐Touriñán et al., 2024), but it is likely the local levels of active BRs—rather than the abundance of BR receptors—that limit signaling. Importantly, BR regulation cannot be understood solely at the cell or tissue scale. At the whole‐plant level, shoot–root interactions add another layer of complexity. Micro‐graft experiments between wild‐type and BR‐null mutants in both Arabidopsis and tomato have demonstrated that shoot‐derived BR can fully rescue mutant root biomass, whereas the absence of shoot BR attenuates root growth (Khandal et al., 2025). Such precise integration of regulatory layers is essential for guiding the complex patterning and growth of the root.

## BR SIGNALING INTEGRATION IN VASCULAR DEVELOPMENTAL PATHWAYS

**Figure 2:**
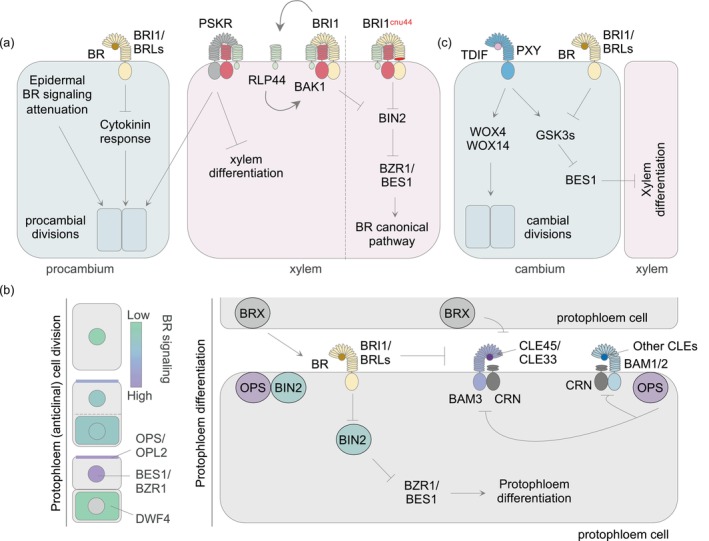
Molecular components of brassinosteroid (BR) signaling and their crosstalk with vascular regulatory pathways. (a) Schematic model illustrating how BR signaling modulates procambial divisions. Through the canonical pathway, BR signaling negatively regulates cytokinin responses. BR‐mediated modulation of procambium activity is regulated non‐cell‐autonomously by BR signaling in the epidermis, as reduced BR signaling in the epidermis can lead to overactivation of BR signaling in the stele. Moreover, BRI1 regulates procambial divisions through a non‐canonical pathway involving RLP44 and the phytosulfokine (PSK) receptor. BRI1 promotes the expression of RLP44, which acts as a scaffold protein enhancing the formation of complexes between the PSK receptor or BRI1 and their co‐receptor BAK1. RLP44 can still form a complex with *bri1* mutant *cnu44* and partially activate the canonical BR pathway but fails to interact with the PSK receptor, preventing regulation of procambial divisions. Although drawn in procambial or xylem cells for clarity, the molecular regulators shown influence both (pro)cambial and xylem fate decisions. (b) In the protophloem, BR signaling is required for both protophloem cell divisions and differentiation. In dividing cells, accumulation of OPS/OPL2 in the upper daughter cells leads to elevated BR signaling, which promotes the expression of cell wall organization‐related genes. In contrast, BR signaling is reduced in the lower daughter cells, where *DWF4* expression is high. BR perception and signaling also regulate protophloem differentiation via the canonical pathway and are hypothesized to antagonize the CLE45‐BAM3 signaling pathway, which represses protophloem differentiation. These processes are regulated by OPS, which modulates BIN2 activity and the formation of the BAM3‐CLV2/CRN receptor complex, and by BRX, which promotes BR biosynthesis and inhibits CLE45‐BAM3 signaling. (c) During secondary vascular development, BRs promote xylem formation in a dose‐dependent manner, whereas TDIF suppresses it via the TDR/PXY signaling pathway. This antagonism likely converges on shared regulators (GSK3–BES1/BZR1), though the involvement of multiple GSKs and BZR factors complicates our understanding of the crosstalk.

In the root apical meristem (RAM), procambial cells divide periclinally to increase the number of vascular cell files, which later differentiate into the conducting tissues, phloem and xylem (De Rybel et al., 2016) (Figure 1b). BRs regulate multiple aspects of vascular development in roots and other organs, from procambial proliferation to vascular tissue differentiation (Furuya et al., 2024) (Figure 2). During primary vascular development, BR biosynthesis and signaling promote cell division to increase the number of vascular cell files and are also required for protophloem differentiation. Part of the regulation of procambial division is mediated through BR‐dependent suppression of cytokinin responses (Ohashi‐Ito et al., 2023), acting via the canonical pathway in a cell‐autonomous manner, as both BR receptors and downstream effectors are present in procambial cells (Figure 2a). However, BR effects on vascular patterning cannot be explained solely by cell‐autonomous and canonical signaling outputs, or at least by the specific molecular mechanism repressing procambial divisions. For example, specifically reducing BR signaling in the epidermis enhances excessive cell division in the stele, whereas loss of BR signaling within the stele itself does not. These findings indicate that BR signaling in the epidermis regulates vascular cell division non‐cell‐autonomously as well (Ackerman‐Lavert et al., 2021). Interestingly, the introduction of BRI1 specifically in the stele of the BR‐blind mutant plants increases the number of vascular cells (Blanco‐Touriñán et al., 2024; Kang et al., 2017). This apparent contradiction may reflect the spatially localized and dose‐dependent effects of BR signaling within the RAM or suggest the existence of alternative regulatory mechanisms. Indeed, BRI1 is also required for root vascular cell fate, as *bri1* null (but not hypomorphic) mutants show ectopic xylem differentiation in procambial positions (Holzwart et al., 2018). Notably, this phenotype appears independent of canonical BR signaling outputs. Instead, BRI1 seems to be required for the expression of its interacting partner RECEPTOR‐LIKE PROTEIN 44 (RLP44), which associates with the receptor of the peptide hormone phytosulfokine (PSK) to promote procambial activity and repress xylem differentiation (Holzwart et al., 2018) (Figure 2a). PSK is a sulfated peptide hormone, that regulates cell proliferation, growth, and various developmental and stress‐related processes in plants (Sauter, 2015). Mutants lacking RLP44 or impaired in PSK signaling display ectopic xylem differentiation in procambial positions reminiscent of *bri1* mutants (Holzwart et al., 2018). These phenotypes are consistent with a model in which RLP44 links BRI1 and PSK signaling, thus providing a mechanistic framework for the dynamic integration of multiple receptor kinase pathways at the plasma membrane to regulate vascular cell fate and patterning. The identification of novel *bri1* alleles has further clarified these dynamic associations (Holzwart et al., 2020). For example, the *bri1* ^*cnu4*^ allele carries a single nucleotide polymorphism resulting in a Gly746Ser substitution within the last LRR of the extracellular domain. This mutation only mildly impairs canonical BR signaling but significantly affects the non‐canonical pathway mediated by RLP44. At the mechanistic level, the *bri1* ^*cnu4*^ mutation sequesters RLP44 in the BR receptor complex, thereby reducing PSK signaling efficiency (Holzwart et al., 2020) (Figure 2a).

BR also directs formative cell divisions and protophloem differentiation in Arabidopsis root meristems (Kang et al., 2017). BR‐blind plants exhibit defects in protophloem differentiation, which can be rescued by protophloem‐specific restoration of BR perception (Kang et al., 2017). This rescue likely involves transcriptional regulation, as single‐cell mRNA sequencing analyses revealed that BR signaling differentially regulates a subset of genes in phloem tissues, including known protophloem regulators, such as *ALTERED PHLOEM DEVELOPMENT* (*APL*), *BREVIS RADIX* (*BRX*) or *CLE26* (Graeff et al., 2021). Moreover, treating Arabidopsis mutants with impaired primary root protophloem differentiation, such as *octopus* (*ops*), with brassinolide partially rescues their differentiation defects (Kang et al., 2017). Pharmacological inhibition of BIN2 with bikinin produced contradictory outcomes, with one study reporting rescue and another not, suggesting that this effect of BR may be dose‐dependent (Crivelli et al., 2025; Kang et al., 2017). These findings are consistent with a model where OPS directly interacts with BIN2 in the presence of BRs, repressing its activity through sequestration at the plasma membrane (Anne et al., 2015; Greenwood et al., 2023; Kim et al., 2023) (Figure 2b). Supporting this view, introducing *bzr1* ^*D*^ or *bes1* ^*D*^ mutations into the *ops* background partially restores protophloem differentiation and overall root growth (Anne et al., 2015), implying that canonical BR signaling components are involved in regulating protophloem development and root growth. We further hypothesize that a feedback loop fine‐tunes this regulation. Bikinin treatment reduces OPS phosphorylation, which may alter its activity, as OPS function is largely determined by its phosphorylation status (Breda et al., 2017; Kim et al., 2023). A recent study has further clarified the role of OPS and its homolog OPL2 in modulating BR signaling within the protophloem (Vukašinović et al., 2025). OPS and OPL2, membrane‐associated and shootward polar localized proteins, accumulate preferentially in the upper daughter cells of the protophloem proliferation domain, correlating with increased nuclear accumulation of BZR1 and enhanced BR signaling (Ruiz Sola et al., 2017; Truernit et al., 2012; Vukašinović et al., 2025) (Figure 2b). Single‐cell transcriptomic analyses reveal distinct cellular states post‐division, with upper daughter cells enriched for BR‐induced genes related to cell wall organization, while lower cells show higher expression of BR biosynthetic genes. Functional perturbation via inducible CRISPR/Cas9‐mediated knockout of *OPS* and *OPL2* disrupts this asymmetric BR signaling, resulting in reduced nuclear BZR1 in both upper and lower daughter cells and elongated protophloem cells that fail to divide (Vukašinović et al., 2025). This OPS/OPL2‐mediated BR signaling activation in the protophloem cells may ensure rapid cell cycle progression and early differentiation.

Protophloem‐specific restoration of BR perception in BR‐blind plants rescues protophloem differentiation defects but also confers partial resistance to exogenous CLE45 and CLE33 peptides (Blanco‐Touriñán et al., 2024; Graeff et al., 2021). Exogenous CLE45/CLE33 application or transgenically increased CLE45 dosage impairs protophloem differentiation, indicating that CLE45 and CLE33 act through an autocrine inhibitory pathway (Carbonnel et al., 2023; Rodriguez‐Villalon et al., 2014). These peptides are primarily perceived by their cognate LRR receptor kinase (LRR‐RK) BARELY ANY MERISTEM 3 (BAM3) (Depuydt et al., 2013) (Figure 2b). Consistent with this, *bam3* loss‐of‐function mutants exhibit specific resistance to CLE45/CLE33‐mediated root growth inhibition, while higher order mutants affecting multiple BAM receptors, such as *bam1 bam3* double mutants, show even stronger and broader resistance to CLE peptides (Hu, Zhu, et al., 2022; Zhang et al., 2024). The CLE45/CLE33 resistance observed in *bri1 brl1 brl3* plants rescued by protophloem‐specific BRI1 expression is BR‐dependent and appears to correlate to some extent with transgene copy number (Blanco‐Touriñán et al., 2024; Graeff et al., 2020). Importantly, this resistance is specific for CLE45 and CLE33 peptides, as these plants remain sensitive to other root‐active CLE peptides, for example CLE26 (Blanco‐Touriñán et al., 2024; Graeff et al., 2020). Moreover, CLE45 resistance was not observed in gain‐of‐function mutants of canonical downstream BR effectors like *bzr1* ^*D*^ or *bes1* ^*D*^, suggesting a model in which BRI1 acts through novel, non‐canonical outputs in the protophloem (Graeff et al., 2020). The molecular mechanisms underlying the antagonistic relationship between BRI1‐dependent BR signaling and CLE45‐BAM3‐dependent pathways remain unclear. However, the direct interaction between BRI1 and BAM3 at the plasma membrane, combined with evidence of interaction between their extracellular domains (Graeff et al., 2020; Smakowska‐Luzan et al., 2018), suggests a compelling possibility: BRI1 may exert a dominant‐negative effect on BAM3 activity (Figure 2b). This would explain both the CLE45 resistance observed in the rescued lines and the protophloem differentiation defects seen in the *bri1 brl1 brl3* triple mutants (Graeff et al., 2020; Kang et al., 2017).

A distinct but not yet fully understood molecular link between BR and CLE signaling has been proposed in other physiological contexts, such as secondary vascular development. Genetic analysis of BR receptor mutants showed an increase in phloem cell numbers and a decrease in xylem cells, mirroring the phenotype of biosynthetic enzyme mutants (Caño‐Delgado et al., 2004; Furuya et al., 2024; Ibañes et al., 2009; Szekeres et al., 1996). Consistently, BR promotes xylem differentiation in a dose‐dependent manner (Kondo, 2022). In contrast, the peptide hormone TDIF, encoded by the *CLE41* and *CLE44* genes, suppresses xylem differentiation during secondary vascular development (Hirakawa et al., 2008). When (pro)cambial cells are simultaneously treated with both TDIF and BRs, the opposing effects on xylem differentiation cancel each other, suggesting a competitive relationship between these signals (Kondo, 2022). Whether this regulation is direct remains unclear, but it was proposed that both pathways converge on the same regulatory module: the GSK3–BZR1/BES1 module (Figure 2c). In contrast to BRs, TDIF activates certain GSK3 kinases, such as BIN2 and BIN2‐LIKE 2 (BIL2), which dissociate from TRACHEARY ELEMENT DIFFERENTIATION INHIBITORY FACTOR RECEPTOR (TDR)/PHLOEM INTERCALATED WITH XYLEM (PXY) receptor kinase, leading to BES1 inactivation (Kondo et al., 2014; Saito et al., 2018). Consistent with this mechanism, inhibition of GSK3 kinases by bikinin promotes xylem cell differentiation (Kondo et al., 2014). These signaling pathways may also interact in the context of mechanical stress. BR signaling mutants (*bes1* and *bzr1*) and plants treated with the BR synthesis inhibitor brassinazole fail to exhibit the typical increase in stem diameter and vascular bundle number following mechanical stress (Raminger et al., 2025). Within the stem, TDIF‐TDR/PXY activates WUSCHEL‐RELATED HOMEOBOX (WOX) transcription factors to promote vascular stem cell proliferation (Etchells et al., 2013). Although BRs promote *WOX4* and *WOX14* expression (Raminger et al., 2025), BES1 has been shown to repress *WOX4* expression (Hu, Hu, et al., 2022). This apparent paradox highlights the complexity of BR‐CLE crosstalk in stems and suggests the involvement of additional regulatory components.

Building on this intricate interplay between BR and CLE signaling pathways, OPS has been identified as a cellular insulator against CLE45 peptide signaling (Breda et al., 2019). Since OPS regulates BR signaling in the protophloem (Anne et al., 2015), it is reasonable to hypothesize that this regulation may be linked to BR signaling as well. However, at least two observations suggest that the role of OPS extends beyond BR regulation: (i) the gap defects in *ops* mutants are more severe than those seen in BR‐blind plants (Kang et al., 2017; Truernit et al., 2012), and (ii) unlike the CLE45/CLE33‐specific resistance observed in *bri1 brl1 brl3* seedlings rescued by protophloem‐specific BRI1 expression, OPS hyperactivity confers resistance to multiple root‐active CLE peptides (Blanco‐Touriñán et al., 2024; Breda et al., 2019; Graeff et al., 2020). OPS may confer this broader resistance by interfering with the interaction between CLE receptors and the putative co‐receptor complex CLV2/CRN (Breda et al., 2019) (Figure 2b). For instance, OPS disrupts the interaction between BAM3 and CLV2/CRN, but this effect is lost when CRN is replaced with a kinase domain‐deleted variant, indicating that OPS targets the intracellular domains to interfere with the BAM3‐CRN interaction (Breda et al., 2019) (Figure 2b). Genetics also revealed that OPS acts downstream of BR perception, since OPS hyperactivity confers CLE45 resistance in the absence of BR receptors (Graeff et al., 2020). Whether OPS contributes to CLE45 signaling regulation by BRI1, and if so, whether OPS functions independently through the mechanisms described, remains to be fully understood. This question is particularly important because we cannot assume that the same mechanism operates in all the regulatory contexts, as the interaction between OPS/OPS‐like and BIN2 appears to be context‐dependent (Anne et al., 2015; Greenwood et al., 2023; Kim et al., 2023; Wallner et al., 2024).

Genetic studies revealed that OPS and BRX interact in regulating protophloem development (Breda et al., 2017). BRX encodes a membrane‐associated protein specifically expressed in the protophloem sieve element precursor cells and functions as a key component of an auxin‐dependent ‘rheostat’ mechanism (Marhava et al., 2018). Like peptide treatments that hyperactivate CLE signaling, loss‐of‐function mutants of *brx* and *ops* exhibit short–root phenotypes with characteristic gaps in the protophloem (Rodriguez‐Villalon et al., 2014; Truernit et al., 2012). These defects are suppressed by mutations in BAM3 or its ligands CLE33 and CLE45 (Carbonnel et al., 2023; Depuydt et al., 2013; Hu, Zhu, et al., 2022), placing BAM3 genetically downstream of both BRX and OPS and suggesting that they act through a shared signaling pathway. While BRX and OPS appear to act in partially parallel branches (Breda et al., 2017), both reduce the ability of the BAM3 complex to transmit CLE45 signals (Figure 2b), either through physical insulation of the receptor within membrane domains (OPS) (Breda et al., 2019) or via modulation of membrane lipid composition and polar auxin flow (BRX) (Marhava et al., 2018, 2020), which in turn conditions cellular sensitivity to CLE signaling. Overstimulation of BR signaling partially rescues the root growth and protophloem gap differentiation defects in *brx* mutants (Rodriguez‐Villalon et al., 2014), indicating that BRX may influence BR signaling. Moreover, BRX participates in a hormonal feedback circuit that fine‐tunes local BR levels (Mouchel et al., 2006) (Figure 2b), potentially interfering with BRI1 activity in the protophloem, and by extension, with the antagonistic BRI1–BAM3 interaction described above. In this way, BRX emerges as an additional integrator of BR and CLE signals in the protophloem, complementing OPS function and contributing to the fine‐tuning of the transition between proliferation and differentiation in vascular tissues.

Collectively, these observations vividly illustrate that BR signaling in vascular development is not a linear, receptor‐to‐transcription‐factor cascade, but rather a multifaceted network of canonical and non‐canonical outputs integrated with other receptor‐mediated pathways, such as PSK, CLE, and auxin signaling. Spatially restricted BR perception, cell type‐specific receptor associations and dose‐dependent effects enable BRI1 to execute distinct functions in the procambium, protophloem, and other vascular cells. Scaffold proteins, such as RLP44, OPS, and BRX emerge as critical molecular ‘signal translators’ that channel BR activity into specific developmental outcomes while modulating the activity of parallel peptide–receptor pairs. Future efforts should therefore be directed toward dissecting the mechanistic logic of these signaling interfaces to fully understand how BRs coordinate with other pathways to achieve the precise spatial and temporal control required for vascular tissue development.

## CONCLUSIONS AND PERSPECTIVES

In this review, we summarized our current understanding of BR signaling specificity. Nevertheless, the precise role of each tissue in mediating BR‐driven organ growth at the cellular level remains somewhat unclear. Systematic analysis using tissue‐specific CRISPR or complementation studies of BR‐blind plants with recoded BR receptor or constitutively active BR effectors such as bzr1^D^ or bes1^D^ may yield complementary insights. Another key aspect in deciphering BR function is understanding the regulatory mechanisms controlling the expression of BR receptors themselves. Expressing a hybrid receptor composed of the BRI1 extracellular domain and the BAM3 intracellular domain under a protophloem‐specific promoter did not result in detectable epidermal signal (Blanco‐Touriñán et al., 2024). In contrast, a hybrid receptor combining the BIR3 extracellular domain with the BRI1 intracellular domain did produce an epidermal signal, suggesting that the CDS of the BRI1 intracellular domain may be responsible for this regulatory pattern (Blanco‐Touriñán et al., 2024). Hybrid constructs between the normal and recoded BRI1 versions present an elegant strategy to pinpoint specific DNA elements driving this regulation. Similarly, the other two BR receptors in Arabidopsis, BRL1 and BRL3, when expressed under a protophloem‐specific promoter, also show weak epidermal expression—a pattern that however is not observed for BRL2, the BRL family member that functions as a medium‐affinity BR receptor (Blanco‐Touriñán et al., 2024; Caño‐Delgado et al., 2004; Caregnato et al., 2025). Yet, recoded versions of BRL1 and BRL3 are needed to unequivocally demonstrate the extent to which their coding sequences influence these expression patterns. Moreover, whether BR receptors in other plant species are governed by similar regulatory mechanisms remains a mystery. Comparative phenotypic analysis of native versus recoded BRI1 constructs under both normal and stress conditions could yield valuable insights into the physiological relevance of this layer of gene expression regulation.

While tissue‐specific knockout of BR signaling or complementation of BR‐blind plants with tissue‐specific BR components is crucial for dissecting tissue contributions to growth, it captures only part of the overall picture. For instance, an unresolved issue is the spatial localization of the hormone itself, as mapping its distribution remains challenging due to its low abundance and the current lack of a suitable fluorescent biosensor (Naqvi et al., 2023; Rowe et al., 2025; Šimura et al., 2018). Moreover, what are the distinct BR signaling outputs in different tissues, and how does BR signaling integrate with other hormonal and peptide signaling pathways to coordinate organ growth? In the root protophloem, for example, the idea that BRI1 antagonizes the genuine CLE45 receptor BAM3 activity remains a hypothesis. Elucidating the molecular mechanisms by which BRI1 exerts a dominant‐negative effect on CLE peptide receptor signaling may provide insights into the crosstalk between these two key ligand‐receptor kinase signaling pathways. At the molecular level, the role of scaffold proteins, such as OPS, BRX, and potentially others in integrating BR signaling with other peptide–receptor pathways during vascular development is still poorly understood and represents an exciting direction for future research.

Finally, integrating emerging technologies such as single‐cell transcriptomics and proteomics (Montes et al., 2024), tissue‐specific genome editing and live imaging will help us understand the dynamic interactions between BR signaling components and elements of other signaling pathways at cellular resolution. Expanding these studies to diverse plant species beyond Arabidopsis will help clarify the evolutionary conservation or divergence of these gene regulatory mechanisms. In sorghum, for instance, recent findings point to a conserved role for SbBRI1‐mediated BR signaling in root development (Rico‐Medina et al., 2025), whereas the BRI1‐ and BZR1‐dependent BR signaling appears to regulate vascular development in *Solanum lycopersicum* and poplar (Jiang et al., 2021; Lee et al., 2019, 2021). Yet divergence remains equally intriguing given that canonical BR perception is thought to have arisen before the divergence of angiosperms, whereas other BR signaling components emerged much earlier (Ferreira‐Guerra et al., 2020; Furumizu & Sawa, 2021; Furuya et al., 2022; Kim & Russinova, 2020; Mecchia et al., 2021). Thus, if BR signaling operates in other than angiosperms, they may rely on yet‐unknown, possibly even nuclear or cytoplasmic BR receptors. Addressing these interconnected questions is key to understanding how BR signaling works not as a uniform growth trigger, but as a modular, locally tuned regulator that coordinates plant development (Boxes 1 and 2).

Box 1 **Bullet‐point summary**

- The coding region of BR receptor genes contributes to their expression pattern.
- BR perception largely functions in a cell‐autonomous manner to regulate root and shoot growth.
- Tissue‐specific BR signaling contributes unequally to overall organ growth, reflecting functional specialization.
- Tissue‐dependent crosstalk between BR perception/signaling and other pathways may contribute to the specificity of organ growth regulation.
- In the root protophloem, the receptor kinase BRI1 may exert a dominant‐negative effect on CLE peptide receptor signaling.

Box 2 **Open questions**

- How are BRs spatially distributed within tissues, and how does this distribution influence local signaling outputs and organ growth?
- What are the specific BR signaling outputs in different tissues, and how does BR signaling integrate with other hormonal and peptide signaling pathways to coordinate organ growth?
- For instance, what molecular mechanisms enable BRI1 to exert a dominant‐negative effect on CLE peptide receptor signaling in the root protophloem?
- How do OPS, BRX and other (un)known scaffold proteins integrate BR signaling with other peptide–receptor pathways to regulate vascular development?
- What is the physiological relevance and molecular mechanism of BRI1 regulation by its own coding sequence, and is this mechanism conserved among BR receptors across plant species?

## CONFLICT OF INTEREST

The authors declare no conflicts of interest.

## Data Availability

Data sharing is not applicable to this article as no new data were created or analyzed in this study.
